# An Integrated Computational Framework for the Neurobiology of Memory Based on the ACT-R Declarative Memory System

**DOI:** 10.1007/s42113-023-00189-y

**Published:** 2023-12-28

**Authors:** Andrea Stocco, Patrick Rice, Robert Thomson, Briana Smith, Don Morrison, Christian Lebiere

**Affiliations:** 1 Department of Psychology, University of Washington, Seattle, WA USA; 2 Department of Psychology, Rice University, Houston, TX USA; 3 Department of Electrical Engineering & Computer Science, United States Military Academy, West Point, NY USA; 4 Department of Bioengineering, University of Washington, Seattle, WA USA; 5 Department of Psychology, Carnegie Mellon University, Pittsburgh, PA USA

**Keywords:** ACT-R, Computational model, Long-term memory, Episodic memory, Semantic memory, Retrieval, Forgetting, Hippocampus, Prefrontal cortex

## Abstract

Memory is a complex process that spans multiple time-scales and stages, and, as expected, involves multiple brain regions. Traditionally, computational models of memory are either too abstract (Shiffrin & Steyvers, 1997) to be meaningfully connected to a biological substrate, or, when explicitly connected, are narrowly focused on one specific region and process (Blum & Abbott, 1996; Weber et al., 2017). By contrast, a comprehensive model of memory with a plausible neural interpretation would be extremely valuable to drive further research in memory function and dysfunction. In this paper, we attempt to fill in this gap by providing a detailed biological analysis of ACT-R’s declarative memory system. This system, developed over four decades, has evolved into a consistent framework that describes how memories are formed, retrieved, forgotten, mistaken, and merged. Building on existing mappings between some components and their biological counterpart, as well as the existing literature, this paper provides a comprehensive view of how the framework’s various computations map onto different brain regions, their network dynamics and functional connectivity, and biological structure. We also show that these mappings provide further insights and explanations for puzzling findings in the memory disorders literature. Finally, we outline the remaining gaps (such as the transition from episodic to semantic memory) and how they could be addressed by future research and modeling efforts.

## Introduction

Long-term memory is a complex mental process that spans multiple timescales and biological mechanisms, from almost-instantaneous synaptic changes at the molecular level to the reorganization of large-scale cortical networks between brain regions taking place over months or years. The complexity and scale of this process pose significant challenges to the computational modeling community. As a result, most of the models that have been proposed cover only a limited amount of long-term memory’s scope. Some models (Shiffrin & Steyvers, 1997) focus on the mechanism of memory retrieval at an abstract level, thus capturing many classic experimental findings but largely avoiding a meaningful connection to its biological substrate. Other models, in contrast, focus on changes in neural activity in a restricted region (for example, the hippocampus: Blum & Abbott, 1996; Weber et al., 2017), successfully capturing neural population dynamics but missing higher-level effects (such as long-term forgetting or the spacing effect) and large-scale neuronal phenomena (such as reconsolidation).

A more comprehensive computational framework of long-term memory, capable of capturing how memories are remembered and forgotten across different temporal scales while also providing a plausible neural interpretation, would be extremely valuable to drive further research in memory function and dysfunction.

In this paper, we attempt to fill in this gap by providing a neurobiological analysis of ACT-R’s declarative memory system. ACT-R is a cognitive architecture (Anderson, 1983a, 2007) and, as such, it encompasses multiple, integrated cognitive components (Anderson et al., 2004). Thus, however, we will restrict our attention to the declarative memory system and consider it independent of the rest of the architecture. Historically, the declarative memory system (Anderson & Bower, 1976) predates the cognitive architecture (Anderson, 1983a)—which is, in fact, built upon it. Moreover, the declarative memory component can be functionally isolated, as demonstrated by the existence of software packages that contain only ACT-R’s declarative memory, with no other component (Reitter & Lebiere, 2010).

ACT-R originated as a mathematical model of forgetting and retrieval, sharing many commonalities with other mathematical approaches to model these processes (Hintzman, 1984; Shiffrin & Steyvers, 1997). Later work, however, has led to a better understanding of the biological bases of the model’s mechanisms (Anderson, 2007; Anderson et al., 2008; Borst et al., 2010) and to a finer interpretation of its equations in terms of neural network dynamics. To the best of our knowledge, most of this progress has been driven by individual contributions focused on specific aspects of the ACT-R memory theory, rather than a global interpretation. This paper intends to fill the gap, tying together the progress made in these different directions into a consistent framework that describes how memories are formed, retrieved, forgotten, mistaken, and merged.

The remainder of this paper is structured as follows. First, an overview of the ACT R memory system is provided. Second, we review the neurobiology of memory at a systems level. Third, we outline a detailed mapping between ACT-R computations and different neural processes. Finally, we discuss a number of new extensions to the ACT-R declarative memory system and their possible biological counterparts.

## The ACT-R Theory of Long-term Memory

The ACT-R theory of declarative long-term memory is the cornerstone of the larger ACT-R cognitive architecture (Anderson, 2007), an integrated computational framework for modeling cognition that is currently, and by far, the most successful and widely adopted architecture in the field of psychology and neuroscience (Kotseruba & Tsotsos, 2020). While the cognitive architecture includes multiple modules that capture sensory, motor, and procedural knowledge, here we will focus on the long-term memory component.

### The Rational Analysis Framework

ACT-R’s declarative memory system was developed within a Bayesian, rational analysis framework (Anderson, 1990). While the ACT-R algorithms can be described without reference to this framework, it is useful to briefly summarize their assumptions. The main tenet of this approach is that memory is shaped by environmental constraints, and thus the availability of a memory reflects the probability that retrieving that memory would be useful at that particular moment (Anderson & Milson, 1989). At the same time, accessing and retrieving memories has a *cost*. An ideal limitless organism (someone akin to the fictional character of Funes in Borges, 1944/1999) would not need to modulate the availability of a memory based on its learned need: every memory would be available at no cost. It is assumed, however, that maintaining memories comes at some price, so that memories should be allocated efficiently, with the most resources invested in the memories that are predicted to be needed (Anderson & Milson, 1989). This acknowledgment that memory is shaped by Bayesian principles but within the constraints of the human brain (i.e., the costs of maintaining memories) makes the rational analysis framework similar to the principle of bounded rationality. If costs are to be interpreted biologically, the same principle is reminiscent of Friston’s (2010) free energy principle, i.e., the idea that the human brain maximizes the predictability of future stimuli (or, equivalently, to minimize their surprisal) with the goal of reducing the metabolic costs.

In rational analysis terms (Anderson, 1990), a memory’s retention function (that is, its availability across contexts and times) adaptively reflects its probability of being needed. Thus, if we indicate the specific memory as *m* and the current context as *Q* (composed of different elemental cues *q*_1_, *q*_2_, …, *q*_*N*_) a memory retention function reflects the logarithm of its posterior need odds *P*(*m*|*Q*)/*P*(¬*m*|*Q*), which can be expressed, per Bayes’ theorem, as the product of prior need odds *P*(*m*)/*P*(¬*m*) and the need likelihood *P*(*Q*|*m*)/P(*Q*|¬*m*). Assuming, for simplicity, that each cue *q* is independent of each other, we can simplify this expression as follows:

1 $$\begin{array}{lll}\log\left(\frac{P\left(\left.m\right|Q\right)}{P\left(\left.\neg m\right|Q\right)}\right)&=&\log\left(\frac{P(m)}{P\left(\neg m\right)}\frac{P\left(\left.Q\right|m\right)}{P\left(\left.Q\right|\neg m\right)}\right)\\&=&\log\left(\frac{P(m)}{P\left(\neg m\right)}\right)+\log\frac{P\left(\left.Q\right|m\right)}{P\left(\left.Q\right|\neg m\right)}\\&=&\log\left(\frac{P(m)}{P\left(\neg m\right)}\right)+\log\prod_q\left(\frac{P\left(\left.q\right|m\right)}{P\left(\left.q\right|\neg m\right)}\right)\\&=&\log\left(\frac{P(m)}{P\left(\neg m\right)}\right)+\sum_q\log\left(\frac{P\left(\left.q\right|m\right)}{P\left(\left.q\right|\neg m\right)}\right)\\&\approx&\log\left(\frac{P(m)}{P\left(\neg m\right)}\right)+\sum_q\log\left(\frac{P\left(\left.q\right|m\right)}{P(q)}\right)\end{array}$$

The last step in Eq. (1) is an approximation derived from the consideration that, for large numbers of memories, *P*(*q*|¬*m*) ≈ *P*(*q*).

The different terms in Eq. (1) have a straightforward explanation in terms of the cognitive psychology of memory (Anderson, 1983b, 1990; Anderson et al., 1996; Anderson et al., 2004). Specifically, the log posterior need odds on the left-hand side of Eq. (1) correspond to a memory’s *activation*, an intuitive construct that describes a memory’s moment-to-moment availability. Similarly, the two quantities on the right-hand side also correspond to well-known cognitive constructs, with the log of the need priors corresponding to the *base-level activation* of *m* or *B*(*m*) (capturing the effects of the prior usage of *m*) and the log-likelihood corresponding to the contextual or *spreading activation* of *m*, or *S*(*m*) (capturing the additive effects that each environmental cue has on the memory’s activation). Thus,

2 $$\begin{array}{lll}A(m)&=&\log\;\left[P\left(m\vert Q\right)/P\left(\neg m\vert Q\right)\right]\\B(m)&=&\log\;\left[P(m)/P\left(\neg m\right)\right]\\S(m)&=&\sum_q\log\left[P\left(q\vert m\right)/P(q)\right]\end{array}$$

and, therefore, *A*(*m*) = *B*(*m*) + *S*(*m*).

### Algorithmic Implementation

To implement these equations algorithmically (Anderson, 2007), the quantities *B*(*m*) and *S*(*m*) are approximated in ways that predict the future use of a memory based on its previous history and its learned associations with contextual cues, respectively. It is this algorithmic implementation that will be used to make contact with neurobiology. Because the algorithmic implementation depends on further assumptions on how memories are represented, it is necessary to briefly introduce the main assumptions that ACT-R makes about the internal structure of memories.

#### Memory Representation

Memories are internally represented as records of features. Historically, these records are referred to as “chunks” and their features as “slots”, although this paper will use the more conventional and less technical terms “memories” and “features”. Features represent the individual, atomic components of a memory, such the basic sensory information (e.g., the color yellow) and elementary concepts (e.g., the magnitude of a number) that make it up. Each feature is identified by a name and a value. For example, the feature of being yellow “yellow” is represented as the pair (Color: Yellow), with the first element being the feature name and the second the feature’s value^1^. Thus, the semantic knowledge that “A canary is a yellow bird” can be represented as a *record* of features such as ((Object: Canary), (Type : Bird), (Color: Yellow)). Memories do not have specific, predetermined types, and a single memory can be made up of an arbitrary number of features. Long-term memory is a finite collection of such memories.

Although this representational format might seem artificial and symbolic, it is, in fact, equivalent to some vector representations used in other models of long-term memory (Hintzman, 1984; Shiffrin & Steyvers, 1997) or in neural network models of semantic (Rogers et al., 2004) and episodic memory (Alvarez & Squire, 1994). In these models, a memory is represented by a vector of fixed size, feature names are represented by a subset of element positions in a vector, and feature values by specific numeric values of the corresponding elements. For example, in Rogers’ model of semantic memory (Rogers et al., 2004), the property (Color: Yellow) is represented in the 64 “perceptual” artificial neurons located in positions 41–104 of the network’s input layer. By contrast, the property (Type : Bird) is represented by values of the 16 neurons in position 137–152. Thus, any ACT-R memory can be transformed into a corresponding vector representation if the list of possible features is predefined and the features that are not present in a memory are set to a default value of zero. This translation scheme, in fact, was used by one of the authors (Lebiere & Anderson, 1993) to create a functional neural implementation of ACT-R. Other encoding schemes that similarly allow for a binding of feature names and values, such as holographic reduced representations (Plate, 1995) or tensor-product variable binding (Smolensky, 1990), have also been used in successful large-scale neural network models of memory (e.g., Eliasmith et al., 2012).

In ACT-R, each memory can be represented only once in long-term memory, and two identical memories cannot exist. Instead, traces of the same memories are indirectly represented by maintaining a list of all of the times the same memory has been re-created and added to long-term memory. The addition of a new trace for a memory, therefore, results in the addition of a new creation time to the list.

### Base-Level Activation, Traces, and Memories

For each memory *m,* its associated base-level activation *B*(*m*) is approximated as follows. In general, researchers agree that the probability of retrieving a memory declines over time according to a power function (Newell & Rosenbloom, 1981). Anderson and Schooler (1991) provided empirical evidence that this power decline over time reflects the statistical properties of the human environment; for example, words in child-directed speech, email receipts, and words in the New York Times titles exhibit similar statistics, with the probability of re-encountering the same word or an email from the same sender decline over time with a power function (Anderson & Schooler, 1991).

This power law applies to every trace associated with a memory. Thus, every time a memory *m* is encoded, a new trace is created, and its availability declines over time according to a power function with a decay rate *d*. The decay rate captures the basic mechanism of “passive” forgetting (Davis & Zhong, 2017), that is, forgetting that is due to the compound effects of different processes such as biological decay (Hardt et al., 2013) or interference from the acquisition of new memories (Anderson & Reder, 1999; Wixted et al., 2004).^2^ The architecture does not make a firm commitment to the precise temporal- or interference-based mechanisms underpinning the decay rate parameter although both are thought to be features of decay (Altmann & Gray, 2002; Lemaire & Portrat, 2018). Note that, separately from the time-based decay captured by the mechanism described in this section, interference from similar memories is also reflected through other mechanisms such as partial matching and blending described in following sections.

The need odds of *m* increase linearly with the number of associated traces. Specifically, because activation is expressed in the form of log odds, the value of *B*(*m*, *t*) at time *t* is the log of the sum of decaying traces associated with *m*:

3 $$B\left(m,t\right)=\log \sum_i\ {\left(t-t(i)\right)}^{-d}$$

in which *t* represents the current time, *t*(*i*) represents the time at which the *i*-th trace has been encoded, the difference *t* − *t*(*i*) is the age of the trace, and the quantity (*t* − *t*(*i*))^*−d*^ represents the declining availability of the *i*-th trace at time *t* (Anderson & Schooler, 1991).

**Fig. 1:**
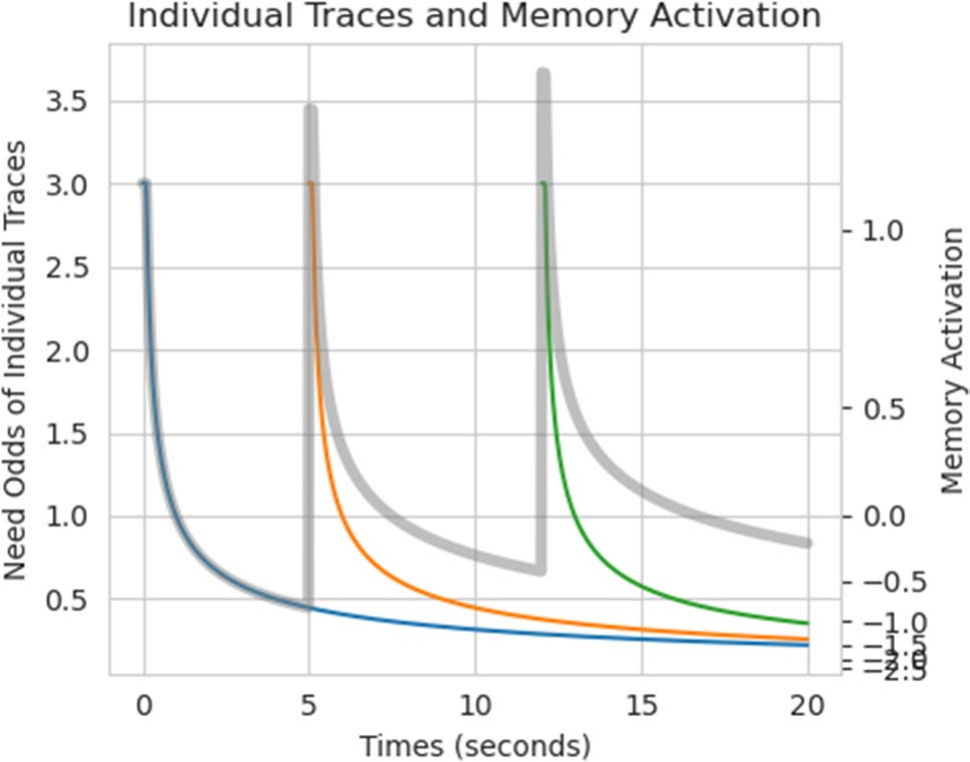
The relationship between a memory *m*’s activation (grey) and the need odds of its constituent traces encoded at times *t*_1_ = 0, *t*_2_ = 5 s, and *t*_3_ = 12 s: blue, orange, and green lines

Figure 1 provides a visual illustration of this mechanism, assuming the same memory has four traces associated with it, generated at times *t*(1) = 0 (memory creation), *t*(2) = 5 s, and *t*(3) = 12 s. Specifically, the colored lines represent the odds of retrieving each trace. Notice the odds of retrieving a specific trace *i* at the time of its creation (that is, when *t* = *t*(*i*)) tend to positive infinity because, at that moment, the probability of *i* being retrieved is exactly 1. Thus, the *y*-scale of the top panel is capped at an arbitrary value of 4. The grey line, on the other hand, represents the memory’s base-level activation *B*(*m*), that is, the log of the sum of each trace’s odds. When *B*(*m*) = 0, the odds of retrieving *m* are exactly 1, implying that *m* is equally likely to be retrieved or not.

#### Recency, Frequency, and the Spacing Effect

Any good memory model should be able to correctly explain the fundamental effects reported in the literature. In addition to the power law of forgetting (Newell & Rosenbloom, 1981), other important memory effects include the recency, frequency, and spacing effect.

**Fig. 2:**
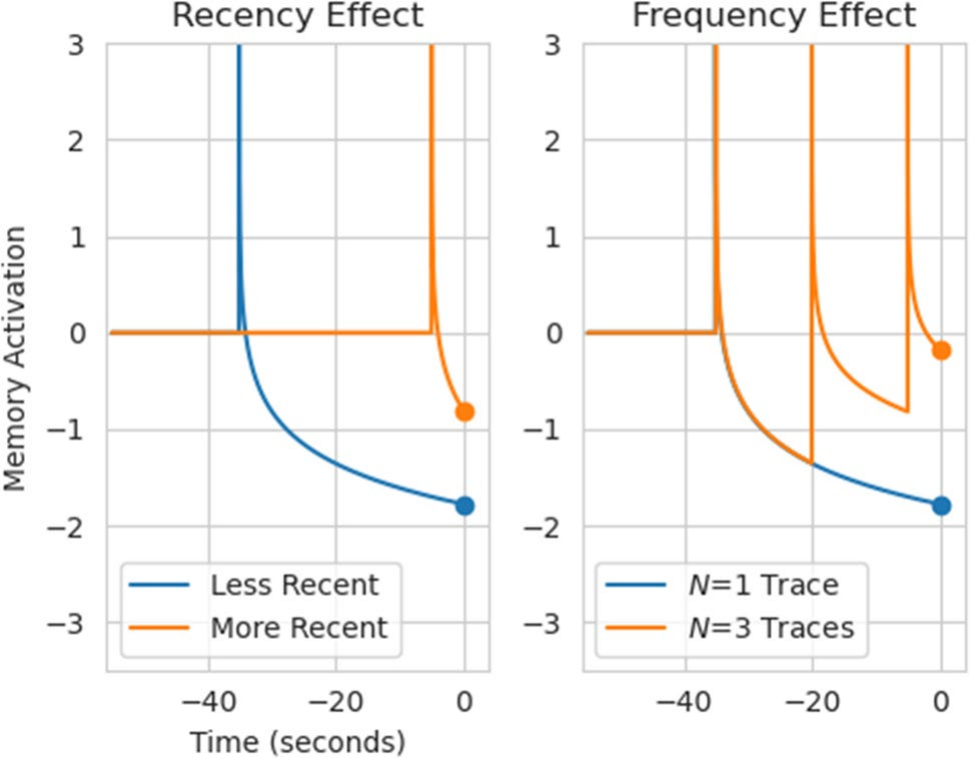
Recency and frequency effects in the ACT-R memory framework. In both figures, time flows from left to right, up to the moment of retrieval at time *t* = 0. (*Left*) In the *recency* effect, the activation of a memory encoded more recently (orange) has declined less than that of a memory encoded earlier (blue). (*Right*) In the *frequency* effect, a memory that has been rehearsed twice after encoding (orange, three traces) retains a higher activation than a memory that has never been rehearsed (blue, one trace)

Equation 3 captures the basic effects of recency and frequency. Recency arises as a consequence of the power law of forgetting, which makes the activation of a memory decline as a power function of its age (Fig. 2). Frequency, on the other hand, depends on the summed effect of the accumulation of traces, by which a memory with more associated traces retains greater activation than a memory with the same age but fewer associated traces (Fig. 2).

In addition to recency and frequency, another fundamental law of memory is the *spacing effect *(Cepeda et al., 2008), that is, the phenomenon by which the probability of retrieving a memory is higher when, all other things being equal, the interval between the encodings of its traces (the “spacing”) is larger. The spacing effect is typically studied in experiments in which a particular item is presented twice, with different intervals between the two presentations; each presentation is assumed to result in an independent trace. The time between the second presentation and the final test is maintained constant, and the interval between the two traces is varied.

It is easy to see that Eq. (3) cannot account for the spacing effect, as the combined effects of each trace are simply additive. If anything, a larger gap implies that the first trace was created earlier than in the case of a shorter gap. Thus, in the case of a larger gap, the activity of the first trace would have decayed more, resulting in lesser activation for the memory—exactly the opposite of what is experimentally found.

To account for the spacing effect, Pavlik and Anderson (2005) introduced a modification to the decay term *d*. Specifically, they relaxed the constraint that *d* is constant across all traces, and allow for every trace to have its own specific decay term *d*(*i*):

4 $$B\left(m,t\right)=\log {\sum}_i{\left(t-t(i)\right)}^{-d(i)}$$

Specifically, the trace-specific term *d*(*i*) depends on the current value of the base level activation *B*(*m*) at the moment at which the trace was created. Thus, when the *i*-th trace is created, it is given a decay rate *d*(*i*) calculated as follows:

5 $$\begin{array}{lll}d(i)&=&ce^{B(m,t=t(i))}+\alpha\\&=&c\sum_{j<{i}}(t-t(j))^{-d(j)}+\alpha\end{array}$$

where *B*(*m*, *t* = *t*(*i*)) represents the value of *B*(*m*) at time *t*(*i*). The spacing effect is made possible by the fact that the term *c* e^*B*(*m*, *t*= *t*(*i*))^ makes a trace’s decay rate dependent on the activation of the corresponding memory at the time of creation. When two traces are temporally close together, the corresponding memory’s activation at the moment the second trace is encoded is higher, resulting in a larger value of *c* e^*B*(*m*, *t*= *t*(*i*))^ and, therefore, a larger decay rate.

The complete model of Eqs. (4) and (5) is noteworthy for its reliability, having been used to successfully model a variety of memory results (Anderson et al., 1999; Pavlik & Anderson, 2005) and having been used to successfully derive optimal schedules for learning practice (Pavlik & Anderson, 2008). The rate of forgetting α in Eq. (5) has been also used as an idiographic (i.e., person-specific) parameter (Sense et al., 2016), with α being a stable and reliable trait within the same individual across sessions and materials, and to assess individual differences in real-life outcomes, such as a student’s success at answering test questions after studying (Sense et al., 2016).

### Spreading Activation and Attention

The spreading activation component *S*(*m*) can best be understood by considering a classic representation format for memories, namely, semantic networks (Collins & Loftus, 1975; Roelofs, 1992). In semantic networks, each memory represents a node, and its components are connected by directional links.

**Fig. 3:**
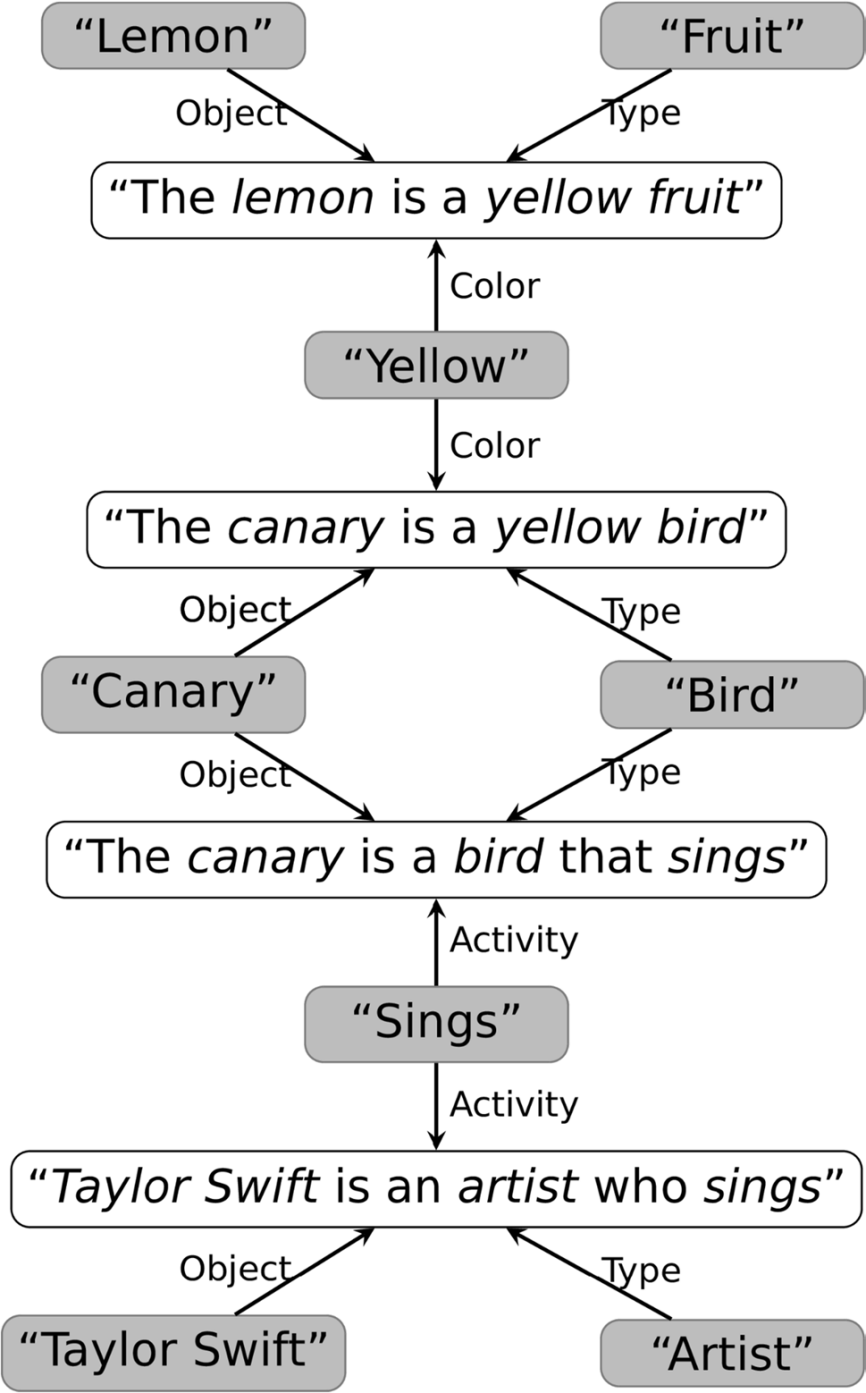
Semantic network representation of the four ACT-R memories “The canary is a yellow bird”, “The canary is a bird that sings”, “Taylor Swift is an artist who sings”, and “The lemon is a yellow fruit”. Gray boxes represent atomic feature values (i.e., terminal nodes in the network), and white boxes represent the memories built upon them

The terminal leafs of this network represent basic, atomic representations, such as the sensory information corresponding to “Yellow” or the abstract concept of “Two”. Figure 3 provides a visual representation of how the concept of “A canary is a yellow bird” is represented in such a network and how its representation partially overlaps with the concept of “The lemon is a yellow fruit”.

In this network, spreading activation is implemented as an additional amount of activation that flows from the memory nodes that are part of the context *Q* = *q*_1_, *q*_2_, …, *q*_*N*_. Thus, each element *q* represents a specific active node in the current context. Specifically, when a particular fact or memory becomes part of the current context, its constituent features become contextual cues *q*_1_, *q*_2_, …, *q*_*N*_ and receive a certain amount of activation that spreads to the immediately connected nodes.

For example, if the context contains the fact that “Lemon is a yellow fruit”, then its features “Lemon”, “yellow”, and “fruit” would receive activation that would spread to all of the nodes that are associated with them. In the case of Fig. 3, only the feature “yellow” is associated with another node (“The canary is a yellow bird”). This node’s base level activation *B*(*m*) will then be increased by the amount of spreading activation *S*(*m*) flowing from the feature “yellow”.

The amount of activation that ultimately reaches a given memory *m* from a cue *q* is scaled by the strength of the link between *q* and *m*. The strength of this link reflects the statistics of co-occurrence between the two events, so that greater co-occurrence of *q* when *m* is present (i.e., *P*(*q*|*m*)) correspond to stronger links. If there is a direct link from *q* to memory *m*, then *m* receives an activation boost that is proportional to the product between the strength of the link connecting *q* to *m* (indicated as *S*_*q*→*m*_) and the attentional weight given that cue. The attentional weight is usually simplified as a single scalar quantity, *W*, that is divided by the number *N* of features that are present in the context *Q*. The total amount of spreading activation *S*(*m*) that *m* receives is the sum of all of the partial effects of each element *q*:

6 $$S(m)=W/N\times {\sum}_{q\in Q}{S}_{q\to m}$$

When *W*/*N* = 1, this equation can be related to the Bayesian definition of *S*(*m*) in Eq. (2) by assuming that each association *s*_*q*→*m*_ approximates the quantity log *P*(*q*|*m*) / *P*(*q*). Note that the denominator *P*(*q*) can be measured by simply counting the number *n* of memories that contain *q* as a feature. Thus, the amount of activation spreading from each *q* is proportional to ≈ log(1/*n*); in other words, it decreases in proportion to how common the feature *q* is across all memories. This assumption plays an important role in explaining some paradoxical phenomena of memory retrieval, most notably the fan effect (Anderson, 1974, 1983b; Anderson & Reder, 1999). In the fan effect, memories that contain more common features (that is, features that show up in many other memories) are less likely to be retrieved than memories that have unique features; this is caused by the denominator *n* being larger for commonly occurring features. The reduction in spreading activation for *high-fan* memories serves as a form of cue interference-based decay.

When *W*/*N* ≠ 1, the parameter *W* can be interpreted as a different weight assigned to contextual vs. base-level information or, in Bayesian terms, to likelihood vs. prior information. In cognitive terms, *W* can thus be interpreted as the amount of attention that is allocated to the current context during retrieval. This form of attentional control can also be interpreted in terms of working memory, that is, an individual capacity to maintain, process, and update short-term information (Baddeley, 1992, 2010; Baddeley & Logie, 1999). Specifically, controlled activation of long-term memory elements through attention can explain the relationship between performance in complex span tasks and the ability to control interference (Burgess et al., 2011; Kane et al., 2001). In fact, Daily et al. (2001) were able to show that individual variations in *W* values capture idiographic differences in working memory performances, and that individual differences in *W* values, when estimated independently through a working memory task, successfully predicted performance on other tasks that demand cognitive control.

Although a skilled modeler might find clever workarounds, ACT-R does not possess a mechanism for separately weighting different cues. The differential weighting might be crucial to capture certain effects in fast memory retrieval (Engelmann et al., 2019). Thomson, Bennati, and Lebiere (Thomson et al., 2014; see also Thomson et al., 2015) integrated a working memory mechanism into ACT-R which adds a short-term decay to memory traces (i.e., chunks cleared from buffers) to provide separately weighted cues based on a degree of residual activation. Models integrating buffer decay have developed a unified account of free and serial recall effects and have been used to study interference effects in memory.

### Retrieval Control, Similarity, and Partial Matching

According to the ACT-R theory, memories are retrieved probabilistically based on the summed effects of their *B*(*m*) and *S*(*m*) components, with memories with higher activation being more likely to be retrieved. In principle, all of the existing memories can compete for retrieval. The set of memories competing for retrieval, however, can also be voluntarily restricted by imposing selection criteria during the retrieval process. These selection criteria are expressed in the form of requirements on the specific features that a memory must have. For example, one could retrieve the common name “canary” by restricting the retrieval set only to those memories that contain the features (Type: Bird) and (Color: Yellow). By specifying appropriate features, one can properly filter out highly active but otherwise irrelevant memories. This form of control over the retrieval process can be understood as a form of executive function (Diamond, 2013; Van der Linden et al., 2000). Unlike spreading activation *S*(*m*), the form of control exerted by selection criteria is driven by internal, possibly voluntary processes and not by learned co-occurrence statistics across memories.

In their simplest form, selection criteria are strictly binary: a memory either contains the specified features (and is thus included in the retrieval set) or not. This is an unrealistic requirement and does not explain errors or intrusions that arise due to the similarity and are extensively documented in memory research (e.g., Mandler et al., 1969). The ACT-R theory allows for such effects by discounting a memory’s activation in inverse proportion to its similarity to the selection criteria. Thus, all memories are included in the retrieval set, independently of the selection criteria or not; however, the less a memory fits the criteria, the more its activation level is penalized and the less likely it is to be retrieved.

Similarity is implemented in the form of a mismatch penalty δ between the value of a feature specified at retrieval and the actual feature value; this value is then added to a memory’s activation. The mismatch penalty represents the dissimilarity between the representation of two values, so that the net effect is to penalize a memory’s activation in proportion to the dissimilarity of its component. Specifically,

7 $$A(m)=B(m)+S(m)+{\sum}_f MP\times \delta \left({f}_r,{f}_m\right)$$

where δ(*fr*, *fm*) is the dissimilarity between the required value of feature *f*_*r*_ and the value *f*_*m*_ of the corresponding feature in memory *m*, the sum is over all features specified in the retrieval, and *MP* is a mismatch penalty scaling factor.

### Blending

In classic ACT-R, a memory *m* is made of identical traces and, once formed, it is never modified. Those identical traces are identified each time one is encountered and merged together, reinforcing the original trace. The retrieval process might occasionally fail, but, when it succeeds, it always produces an accurate copy of a memory.

Although these assumptions greatly simplify the computational process, they also contradict several known aspects of human memory. First, no two identical traces are made; in fact, the MTT assumes that similar traces are aggregated, and that this aggregation process eventually produces the abstract categories of semantic memory (e.g., the knowledge of what a “bird” is). This phenomenon is the key to extract general features from examples and to develop prototypes (Rosch & Mervis, 1975).

Second, retrieval is a reconstructive process. That is a direct consequence of how memories are implemented in the brain, with the retrieval of a memory causing a reactivation of the original brain state at the moment the memory was encoded (Danker & Anderson, 2010).

To overcome these limitations, ACT-R allows for a special mechanism known as *blending *(Lebiere, 1999). Blending allows one to retrieve memories that are a mixture of features in the pool of relevant memories, while not being exactly identical to any of them. Specifically, the newly retrieved blended memory will possess a series of features, and the blended value f* of each feature will be calculated as to minimize its dissimilarity with the homologous features *f*_*m*_ of every other memory *m* that is being considered for retrieval:

8 $${f}^{\ast }={\textrm{argmin}}_f{\sum}_mP(m)\times \left[1-\textrm{sim}{\left(f,{f}_m\right)}^2\right]$$

where *P*(*m*) is the probability of retrieving a given memory *m*. In turn, *P*(*m*) depends on the current activation of *m*, thus scaling the importance of each feature value by the activation of its memory.

This formulation generalizes at least three distinct cases. If the feature values are numerical, then the blended value is effectively the average of the memory features, weighted by each memory’s probability. The type of average depends on the similarity function: if similarities are linear, i.e., proportional to the difference between two values, then the blended value corresponds to the arithmetic mean, whereas if similarities reflect the log ratio of the largest to the smallest value, then the blended value corresponds to the geometric mean. The second case, which is the opposite condition to the first, corresponds to symbolic values that are maximally dissimilar to each other (and maximally similar to themselves). In that case, blending corresponds to weighted voting, in which the feature chunk with the largest summed probability over all memories is selected. The third and most general case between these two extremes applies to feature chunks that have similarities to each other, which can be understood as corresponding to vector embeddings within a high-dimensional space. In that case, the blended value can be a feature chunk that is not the most common but instead represents the best weighted compromise between all feature values.

Note that, in theory, blending produces new memories that average across different memories each of which, however, is still made of identical traces. However, it is possible to create “episodic” chunks that have only one trace associated with them; in this case, the blending mechanism becomes equivalent to the averaging over traces that occurs in the multiple-trace theory, and constitutes the general case to the merging of identical memories.

Blending has been successfully used in Instance-Based Learning (IBL: Gonzalez et al., 2003), a theory of experiential decision making that posits that decisions are made by sampling from memories of past outcomes in similar situations. Unlike other decision-by-sampling approaches, which posit that multiple retrievals are made (Stewart et al., 2006), IBL relies on blending to retrieve a single memory that is prototypical of the previous outcomes of a decision.

## Neurobiology of Long-term Declarative Memory

Before attempting to map the different facets of the ACT-R memory system to their putative neural substrate, it is important to outline what is the general consensus view on the neuroscience of memory. For simplicity, we will articulate this consensus view according to the life cycle phases of a memory, that is, encoding, consolidation, and retrieval.

### Memory Encoding

The lifetime of a memory begins with the process by which it is encoded as a representation in the neural tissue. Such a representation, which is often referred to as the “engram” (Josselyn et al., 2015), represents the biological equivalent of a “trace” in ACT-R and in the Multiple Trace Theory—namely, the earliest possible stage at which a memory exists.

A series of landmark studies of amnesic patients have provided conclusive evidence that the medial temporal lobe, and, specifically, the hippocampus, plays a critical role in the creation of declarative memories. Patients suffering from bilateral damage to the hippocampus invariably present a deep form of amnesia, which provides a connection between this particular brain region and long-term memory (Corkin, 2002; Gabrieli et al., 1988; Scoville & Milner, 1957).

Note that *non*-declarative memories are spared by hippocampal damage. Multiple studies have shown that, even in deep forms of amnesia, memories for habits and skills (Knowlton et al., 1996) and implicit memories (Schacter et al., 1993) remain intact. The scope of memory impairment following hippocampal damage amnesia, however, perfectly overlaps with the scope of the ACT-R’s declarative framework, which encompasses both semantic and episodic memories.

The physiological organization of the hippocampus provides a clue about the nature of its representations. The hippocampus receives a topologically organized projection from the cortical mantle. Internally, the hippocampus consists of massively interconnected projection neurons. This organization is remarkably similar to that used in artificial neural networks known as auto-associators (Hopfield, 1982; Treves & Rolls, 1994), which are capable of quickly learning new patterns of inputs that can be internally stored in the different strengths of synapses. Indeed, the original Hopfield model, which is capable of one-shot learning through the simple and biologically plausible Hebbian rule, remains to this day an effective and useful model for hippocampus learning and deterioration (Weber et al., 2017).

### Storage and Consolidation

Although patients who suffer from bilateral medial temporal lobe damage are unable to form new memories, they typically retain memories of past events, a characteristic pattern known as anterograde amnesia. This suggests that although the hippocampus is required for creating new memories, it is not the ultimate repository of memories—otherwise, all memories would be lost following bilateral hippocampal damage. In turn, this suggests that a memory’s representation (its engram) changes over time.

But how and where are memories ultimately stored? Multiple lines of evidence suggest that memories might be ultimately represented as massively distributed patterns of synapses between neurons located in different areas of the neocortex. This distributed organization explains why retrograde amnesia (that is, the loss of previous memories accompanied by the ability to form new ones) is exceedingly rare, and no known focal brain lesions give rise to it (Hardt & Sossin, 2020). If the cortex is the ultimate seat of long-term memory and the hippocampus is the first, then a transfer process must occur that moves information from the hippocampus to the cortex over time. This transfer process is known as systems consolidation.

The existence of systems consolidation is also suggested by the fading pattern of memory loss in amnesic patients. In these patients, anterograde amnesia is typically accompanied by a gradient of retrograde amnesia, with the depth of memory loss being larger in proximity of the time at which the hippocampal lesion has occurred and extending, in increasingly small degree, as far as a couple of years in the past. This pattern has been taken to imply that systems consolidation takes place over multiple months or years, during which the hippocampus and the neocortex balance the load. An influential model analysis (McClelland et al., 1995) proposes that this is due to the difference in the durations of learning in the two structures, with the neocortex being specialized for long-term, slow learning and the hippocampus for fast, one-shot, quick learning. The difference in size between the hippocampus and the cortex also suggests different limitations in capacity, with the smaller hippocampus better suited to providing a fast way to store memories for a limited amount of time. Alvarez and Squire (1994) proposed an early influential model of how such transfer happens. In the model, the encoding of memory happens in parallel at two levels, the cortex and the hippocampus, following two different time courses. The hippocampus forms fast associations between local populations of neurons activated by corresponding cortical projections. When a partial pattern of a previously encoded memory is present in the cortex, the hippocampus is thus capable of re-activating all of the previously active neurons, which in turn re-activate the original cortical sources through the ascending fibers that proceed from the hippocampus to the cortex. Thus, the fast associations learned by the hippocampus act as a bridge, allowing the re-activation of a large ensemble of cortical neurons given a partial representation. This process is analogous to the retrieval of a memory given a cue. Over multiple retrievals, co-activated neurons in the cortex form stable synaptic bonds with each other and, as a result, can recreate full patterns from partial cues without the help of the hippocampus. In fact, it has been speculated that the cortex and the hippocampus have complementary roles and learning mechanisms (McClelland et al., 1995). In recent years, imaging experiments have provided detailed evidence that semantic memory is distributed across all cortical regions, forming a continuous, multidimensional semantic space (Huth et al., 2012; Mitchell et al., 2008).

Although the general theory of systems consolidation is universally accepted, two different variants exist. The difference between these two frameworks concerns the different involvement of the hippocampus in semantic vs. episodic memories, that is, first-person, autobiographical memories of real-life events (Tulving, 1993; Tulving et al., 2002). In the so-called Standard Model of Consolidation, both semantic and episodic memories are ultimately transferred from the hippocampus to the cortex. According to the Multiple Trace Theory (MTT), however, episodic memory traces remain in the hippocampus, while semantic memories are slowly transferred to the cortex (Moscovitch et al., 2005; Nadel et al., 2000). Evidence for the MTT comes from many neuroimaging studies that show that the retrieval of episodic memories, but not of semantic ones, is associated with surges of activity in the hippocampus (Cabeza et al., 1997; Moscovitch et al., 2005).

In fact, the retrieval of episodic memories involves the hippocampus and a number of additional brain regions that are connected to it. These regions form a functionally interconnected network known as the Default Mode Network (DMN: Raichle, 2015). The DMN has been the focus of much research in the past decade, since it was discovered that it is active during spontaneous thought and inhibited during task-related and goal-directed activity—one of the reasons why it had been originally overlooked in neuroimaging (Raichle et al., 2001). Although the exact nature of this spontaneous brain activity is unknown, a dominant hypothesis is that it serves to maintain stable patterns of connectivity that encode Bayesian priors, shaped by previous experiences, about the type of information that the brain is likely to encounter in the future (Pezzulo et al., 2021).

Note that, although the distinction between episodic and semantic memory is critical in the neurosciences, it is not mirrored in ACT-R, which currently does not distinguish between the two. Because of this, different researchers have used ACT R’s mechanisms and representations for either purely semantic memories (e.g., the multiplication tables in arithmetic: Rosenberg-Lee et al., 2009) or purely episodic memories (e.g., intrusive memories of traumatic events; Smith et al., 2021), as well as complex mixtures of knowledge (e.g., syntactic and semantic information in the mental lexicon; Lewis & Vasishth, 2005). Suggested mechanisms to formalize this distinction into ACT-R are reviewed in the “Episodic and Semantic Memory” section.

### Forgetting

Forgetting is the name given to a variety of processes that counter the effects of consolidation and weaken memories over time. Like memory itself, forgetting is a complex process that involves a variety of mechanisms at different levels (Davis & Zhong, 2017). At the molecular level, forgetting has been found to be associated with at least one specific protein RAC1, the expression of which at the synaptic level induces memory forgetting in animal models (Shuai et al., 2010). Because the expression of RAC1 is triggered by learning itself, it has been speculated that it is selectively involved in weakening existing engrams to facilitate the encoding of new ones (Davis & Zhong, 2017).

If the loss of synapses (as that induced by RAC1) damages a memory’s engram, so does the loss of pre- and postsynaptic cells. Thus, unsurprisingly, forgetting might also happen at the cellular level because of neuronal death (technically known as apoptosis). This is perhaps most dramatically apparent in neuropathologies such as Alzheimer’s disease and other dementias that affect the temporal lobe, and in which the loss of temporal gray matter parallels the depth and severity of amnesia.

Loss of cells and synapses can be seen as specific examples of the general process of biological decay. Other manifestations of biological decay processes include, at the molecular level, the loss of protein kinase C isoform M-zeta (PKMζ), which is necessary to support existing synapses and long-term potentiation. To a certain extent, decay of existing engrams is a necessary part of the process of systems consolidation, since the “transfer” of memories from the hippocampus to the neocortex necessarily implies that hippocampal representations can be erased (Hardt et al., 2013).

Finally, and at a different end of the spectrum, forgetting could be viewed not as the result of a weakening of a memory’s engram, but as the result of interference due to the accumulation of memories since the first trace was encoded (Anderson & Neely, 1996). According to this view, engrams are not lost, but they become progressively inaccessible, and forgetting is the manifestation of a retrieval problem. Dramatic evidence in this sense comes from studies that have shown that chemical or electrical stimulation of the brain often results in vivid flashbacks of long-forgotten memories (Penfield & Perot, 1963), although the reliability of such accounts has been called into question (Loftus & Loftus, 1980), and from rare reports of sudden retrieval of long-lost memories in amnesic patients (Lucchelli et al., 1995).

### Retrieval

In most cases, forgetting can only be inferred as the complement to the process of successfully retrieving memories. In the case of declarative memory, retrieval is accompanied by the conscious re-experience of the event and, at the neural level, by the re-activation of the original patterns of sensory information in the cortex (Danker & Anderson, 2010).

The retrieval of declarative information can happen spontaneously, with minimal attention and effort. However, under many circumstances and especially in laboratory conditions, the retrieval of memories is a controlled and effortful process. A specific portion of the ventrolateral prefrontal cortex is associated with controlled memory retrievals (Badre & Wagner, 2007). In neuroimaging studies, experiments that manipulate the difficulty of retrieval also produce a corresponding increase in the metabolic activity of the VLPFC. The difficulty of retrieval can be manipulated either by increasing the competition between possible retrieval targets (Thompson-Schill et al., 1997), by decreasing the frequency of the target items (Danker et al., 2008) or by increasing the fan between the retrieval cue and the target memory (Danker et al., 2008).

Interestingly, lesions in this region are also associated with *confabulation*, a neuropsychological disorder characterized by incoherent memories. Patients who confabulate tend to provide fictional and often imaginative answers to autobiographical questions, and are apparently unable to realize the erroneous nature of their recollections. Studies have shown that confabulation is associated with specific deficits in the strategic control of retrieval. Specifically, confabulations are associated with specific failures in monitoring the content of retrievals, and are thus associated with greater false alarms in memory judgements as well as inflated confidence in their own accuracy (Gilboa et al., 2006).

## Neurobiological Mapping of the ACT-R Declarative Memory Model

ACT-R’s declarative memory system exists within the larger ecosystem of the ACT-R architecture (Anderson et al., 2004), a comprehensive formal theory of cognition whose development has also been guided by neuroscientific findings (Anderson, 2007; Anderson et al., 2008). Therefore, before outlining the mapping between the different facets of ACT-R’s declarative memory and the neurobiological circuits that support long-term memory, it is useful to first summarize the work that has already been done to map individual modules of the ACT-R architecture onto brain circuits.

### Existing Mapping of the ACT-R Architecture to Brain Regions

**Fig. 4:**
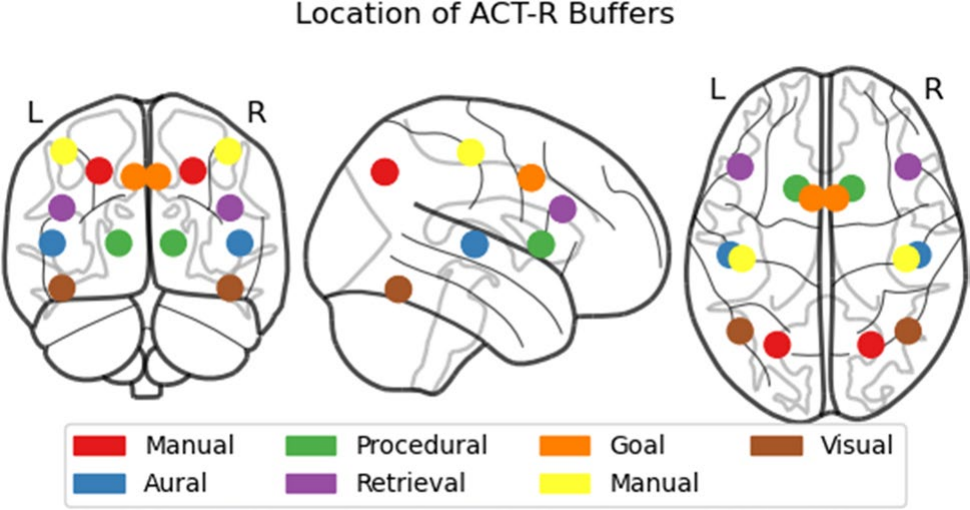
Locations of the ACT-R’s buffers on the outline of the MNI template brain

The ACT-R architecture contains several modules, that is, specialized components that carry our specific, fundamental cognitive functions. The declarative memory system is one such module; others include a procedural module for long-term procedural memory, an imaginal module to manipulate internal representations, a goal module to hold internal representation that solve potential conflict between responses, and a variety of modality-specific perceptual and motor modules. Each module contains one or more buffers, i.e., locations where feature-based representations are placed in order to be operated upon. Each buffer can hold only one representation (that is, only one collection of features or one “memory”); the operations that occur on that representation are module-dependent. Figure 4 illustrates the putative location of the regions corresponding to ACT-R’s main buffers.

Buffers are important to ACT-R’s memory system because representations enter (or re-enter) long-term memory only through a buffer. For example, upon seeing a canary, the contents of visual perception first enter the system in the form of the feature-based representations [(Name: Canary) (Type: Bird) (Color: Yellow)]. It is only after this representation is no longer used by the visual buffer (for example, because a new object enters the visual field, or because attention shifts to a different modality) that it is added to long-term memory. If no previous memory existed with these features, a new memory is created. If an identical memory already existed, a new trace is added to the existing memory, thus increasing its activation (per Eq. (3)).

Buffers are the only structures of ACT-R that have been spatially connected to specific portions of the cortex. Memories and their features do not have a natural interpretation in terms of different populations of neurons, but buffers do. The interpretation of buffers as localized cortical areas was also assumed in the Conditional Routing model (Stocco et al., 2010), a large-scale neural-network implementation of the basal ganglia system that was shown to be compatible with ACT-R’s procedural module.

All of the existing buffers in ACT-R are associated with neocortical regions in the parietal (imaginal buffer), temporal (visual buffer, auditory buffer), and frontal lobe (retrieval buffer, manual buffer, goal buffer). These buffers cover only a small portion of the cortex; other cortical regions could be interpreted as belonging to the modules (rather than the buffers), or to other components that are either hypothesized or not included in the architecture (as the Emotional Module; Juvina et al., 2018) or as providing specialized communication pathways between two other areas (a form of procedural knowledge not dependent on the basal ganglia; Rice & Stocco, 2019).

Although their functional roles are different and dictated by the specific computations of their modules, all buffers share the common characteristic of temporarily holding memories. In the most general terms, a buffer thus contains a collection of features, which are specific to the functions of the underlying module. Thus, buffers in sensory and perceptual modules will typically contain sensory and perceptual features (that is, memories of sensory and perceptual events), and motor modules will contain motor features (that is, memories of motor commands). Thus, the distributed nature of semantic information (Huth et al., 2012; Mitchell et al., 2008) is reflected in the spatial distribution of ACT-R buffers.

**Fig. 5:**
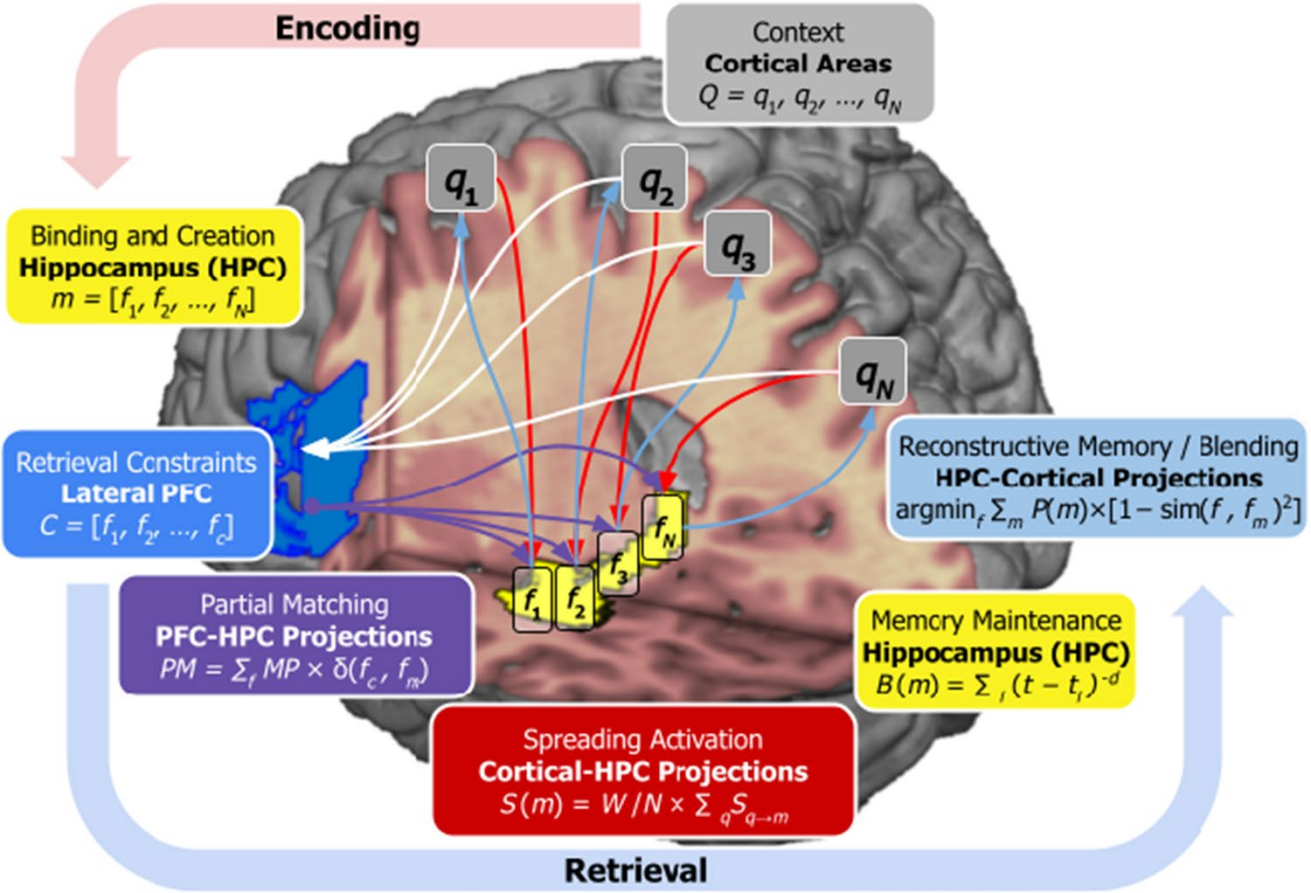
Overview of the mapping between brain architecture and the ACT-R model of long-term memory. During encoding (red arrow), individual features *f*_1_, *f*_2_, … , *f*_*N*_ from different cortical regions (grey) are bound together into a new memory in the hippocampus (yellow). During retrieval (blue arrow), the joined forces of constraints held in the lateral PFC (blue), similarity across memories (purple) and spreading activation from contextual cues *q*_1_, *q*_2_, …, *q*_*N*_ , and the degree of activation of competing memories (yellow) lead to the selection of a recalled memory. The retrieval of information allows the remembered features, potentially blended with attributes from the competing memories, to be transferred back to the corresponding cortical regions (light blue)

Of the existing buffers, the retrieval buffer is the only buffer associated with declarative long-term memory and also the only aspect of declarative memory that has a localized, agreed-upon component in the lateral PFC (Anderson, 2007). The goal of this paper is to provide a finer-resolution mapping of the individual components of ACT-R’s declarative memory module. Figure 5 provides an overview of this mapping, while the following sections will further clarify its rationale.

### Memory Encoding, Feature Binding, and the Hippocampus

In the brain, memories are created when incoming information from cortical regions excites cells in the hippocampus, in turn triggering associative learning between the active hippocampal cells. This mechanism is reflected in ACT-R, whose only way to add memories to the long-term memory store is through the contents of buffers, which are themselves associated with cortical regions (see Fig. 5). In fact, a Hopfield-like model of the hippocampus was incorporated in an neural network implementation of ACT-R to store declarative memories from incoming cortical patterns (Lebiere & Anderson, 1993).

It has been already noted that the ACT-R’s concept of memory implies multiple forms of binding, not only between each feature and its value but also between different features. This binding is the characteristic of autoassociators and, indeed, their main function. Similarly associated with the hippocampus is the way in which ACT-R maintains the count of multiple traces.

As noted in the “Memory Representation” section, in ACT-R, the internal structure of a memory (that is, the number and types of its features) does not affect its basal-level activation *B*(*m*) and its temporal decay *d*. In other words, all memories take up the same biological resources. In reality, however, the internal representation of a memory plays an important role in shaping its future life. For example, items encoded in the same list are often recalled out of place (Lee & Estes, 1977), and target words in a list are if similar-sounding words are read aloud before testing (Oberauer & Lange, 2008). This fact suggests that similar features tend to overwrite each other during encoding, a process that has been likened to the interference that occurs when training neural networks (McCloskey & Cohen, 1989). Furthermore, low-frequency words tend to be remembered less than high-frequency words (Diana & Reder, 2006), suggesting that experience affects the encoding process, Similarly, Alvarez and Cavanagh (2004) showed that items made of either very complex features (i.e., Japanese characters) or containing overlapping features (e.g., identical cubes with different shadings) take up more cognitive resources than simpler items (e.g., color patches). Specifically, items with more complex or overlapping features took longer to find in a visual search task and fewer of them can be held in visual working memory, implying greater representational or processing demands.

Note, although ACT-R has no agreed-upon mechanisms to capture interference during encoding, it does allow for modeling interference at the time of retrieval through the mechanisms of partial matching (see “Retrieval Control, Similarity, and Partial Matching”) and blending (see “Blending”). In both cases, a memory becomes less likely to be retrieved because of the competition of similar memories.

### Base-Level Activation and Forgetting

In a model autoassociator like a Hopfield network, identical states that are memorized result in a linear increase of the strength of the corresponding synapses (Hopfield, 1982; E. Rolls & Treves, 1997), thus increasing the likelihood of their retrieval. This is because, when using the Hebbian learning rule, each time the same pair of neurons is active, the weight of the synapse between them grows by a fixed amount. This behavior is consistent with ACT-R’s mechanism of considering identical copies of a memory as identical traces, and summing the contribution of each trace.

However, unlike traditional associators, ACT-R includes a trace-specific decay parameter *d*(*i*) to the base-level term *B*(*m*), which in turns depends on the rate of forgetting term α in Eq. (5).

The trace-specific decay parameter captures the observed regularity in testing data known as the power law of practice and forgetting (Anderson & Schooler, 1991; Newell & Rosenbloom, 1981): namely, memory performance worsens as a power function of the time elapsed. This regularity was first observed by Ebbinghaus himself (Ebbinghaus, 1885/2013), in the very first experimental study of long-term memory. Decay parameters have been included in multiple memory models; notably, one such parameter explicitly included in Alvarez and Squire (1994)’s foundational neural model of the hippocampus and and in Loftus’ (1978) seminal re-analysis of recall accuracy data. An open debate, which has gone on for decades, is whether the power law of forgetting reflects some form of biological decay or is simply the reflection of interference due to the accumulation and prioritization of traces (Farrell et al., 2016; Hardt et al., 2013; Ricker et al., 2020). Depending on the particular task, evidence has been found for both theories, with neither theory definitively explaining all extant data. Trace decay can result in simple autoassociators due the fact that other memories sharing the same synapse might take over the retrieval process, and this has been found in spiking models of neural populations, which mimic both temporal and interference-based trace decay based on a tradeoff in memory precision (Bays, 2014; Oberauer et al., 2016).

#### The α Parameter and Forgetting

In general, it is possible to see the rate of forgetting α as a computational term that encompasses multiple biological processes involved in forgetting (Davis & Zhong, 2017). These processes include molecular mechanisms, cell death, decay, and interference. These mechanisms do not include, however, some of the “active” mechanisms of forgetting, such as retrieval-directed forgetting and motivated forgetting. To the best of our knowledge, these phenomena are outside the scope of the ACT-R theory of memory.

This biological interpretation of α entails that individual differences in the rate of forgetting would also be reflected in individual differences in neural activity. Experimental evidence for this prediction comes from a recent study by Zhou and colleagues (Zhou et al., 2021). Using an adaptive fact learning paradigm developed by van Rijn and colleagues (Sense et al., 2016), the authors measured the specific value of the α parameter for fifty healthy participants, and correlated individual differences in this parameter with individual differences in the resting state EEG power spectrum, which is known to capture stable individual differences in neural activity. Consistent with the interpretation of the decay rate as a distributed parameter, the authors found that the rate of forgetting α was not singularly localized and was reflected in the power value of multiple scalp locations and frequency bands, implying that the rate of forgetting was related to spontaneous activity of cortical circuits.

Conversely, if the α parameter captures the biological mechanisms of forgetting, then it should be abnormally high in populations that suffer from abnormal memory processes. In fact, a recent paper showed that individuals suffering from amnestic cognitive impairments do exhibit significantly higher values of α than healthy control when their data is modeled using Eqs. (4) and (5); in fact, the value of the α parameter alone was sufficient to separate patients from controls with > 80% accuracy (Hake et al., 2023).

### Spreading Activation and Cortical Projections to the Hippocampus

ACT-R’s spreading activation term *S*(*m*) reflects the effect of distributed representation of the current context *Q*; in ACT-R, the spreading activation component of a memory *m*’s activation is sourced from the contextual features *q*_1_, *q*_2_, …, *q*_*N*_ represented by each buffer in the system (Fig. 5). An individual buffer is capable of providing a scalar amount of activation *W*, divided across *N* features in the buffer, so that a memory receives a portion of this activation for each feature it shares with the buffer’s representation. The allocation of attention *W* contributes to the strength of contextual retrievals. In this manner, ACT-R allows for context to have an effect on memory activation, and subsequently likelihood of retrieval.

The conceptualization of buffers as localized cortical areas carries the implication that a buffer, in neurobiological terms, consists of a limited population of neurons. Representation of multiple features within a limited pool is supported by the formation of representational ensembles, through co-activation of subsets of neurons within the population (Miller et al., 2014). Individual neurons can participate in multiple ensembles, and top-down influences such as attention result in greater recruitment of neurons into representational ensembles (Murray & Wojciulik, 2004; Xin et al., 2019). The set of neurons contributing to a representational ensemble defines the amount of spreading activation *W* that is able to be supplied by that representation; however, as the number *N* of features represented by the population increases, the fidelity of representation of any given feature is degraded (Bays, 2014). This degradation corresponds to a reduction in the activation that can be spread from the cortical population to hippocampal networks sensitive to a given feature, as less cortical activity is dedicated to representation of this feature.

While the amount of “source” activation *W* that is available to be spread is dependent on the extent of cortical representation, connectivity between these cortical populations and downstream hippocampal regions can filter how much of this activation is spread to a given memory. In ACT-R, the amount of spreading activation that a memory *m* receives from a given feature *q* is modulated by the strength of association between *q* and *m*; as stated previously, this associative strength *S*_*q→m*_ reflects the statistics of co-occurrence between the feature and the memory. Per the Hebbian learning rule, this perspective is identifiable with the extent of synaptic connectivity between individual neurons in neocortical and parahippocampal populations. The dual stream model of cortico-hippocampal connectivity proposes that “what” representations originating from temporal cortex are routed to the hippocampus through the perirhinal cortex and lateral entorhinal cortex, while “where” representations formed in the parietal cortex project to the parahippocampal gyrus and then medial entorhinal cortex before encoding in the hippocampus itself (Burwell, 2000). Under this schema, projections from the hippocampus back to the entorhinal cortex implement memory retrieval into the neocortex (Rolls, 2018). However, recent evidence demonstrates direct cortico-hippocampal connections, including connectivity between hippocampus and temporal, parietal and early visual cortices (Huang et al., 2021); it has been proposed that these direct connections could aid in recollection of information from the hippocampus via backprojection to superficial layers of neocortical areas (Huang et al., 2021; Rolls, 2021).

Due to the complexity of connections between neocortical areas and the hippocampal system, ACT-R is ambivalent as to the exact synaptic sites that are modeled by *S*_*q→m*_. Cortical populations involved in representation of commonly-encountered features that are prevalent in memory would be expected to have stronger synaptic connections with hippocampal circuits which encode memory of the same features, compared to less common stimulus features. However, as these common features are overlearned, the extent of the cortical ensemble necessary for representation may be reduced (Dudai et al., 2015); while this would result in less source activation *W*, greater associative strength *S*_*q→m*_ with hippocampal populations would counterbalance this effect.

### Ventro-Lateral Prefrontal Cortex, Retrieval Control, and Retrieval Cues

As noted above, access to declarative knowledge can be spontaneous or controlled, and controlled access to long-term memories is believed to be mediated by the VLPFC (Badre & Wagner, 2007; see Fig. 5). Not coincidentally, in the conventional mapping of ACT-R modules (Anderson et al., 2008) to the brain, the VLPFC is put into correspondence with the retrieval buffer. As noted in the “Retrieval Control, Similarity, and Partial Matching” section, the retrieval buffer functions by holding a specific subset of features that aid in the selection of memories to be retrieved.

A plausible interpretation is that, at the biological level, the VLPFC supports retrieval by holding temporary representations of these features (Danker et al., 2008). In the canonical interpretation (Anderson et al., 2008), a cortical region’s BOLD response is proportional to the amount of time the corresponding buffer is busy holding a set of features. In the case of the retrieval buffer, this provides an elegant explanation for the observed responses of the VLPFC: its BOLD activity in fMRI studies would be driven by any factor that slows down retrieval times, which includes competition between responses (Thompson-Schill et al., 1997) but also the frequency of the study item (Danker et al., 2008). There are at least two possible pathways by which retrieval features could be delivered to VLPFC. One is through the large series of cortico-cortical connections that project to it from other cortical regions, shown in white in Fig. 5. The second is through thalamic inputs gated by the basal ganglia (Scimeca & Badre, 2012; not shown in Fig. 5). The theory does not distinguish between the two, although the latter pathway is compatible, within the larger architecture, with the necessary role played by procedural knowledge in transferring information between buffers.

Within ACT-R, the type of control exerted by retrieval cues is different than that achieved through spreading activation. Specifically, while spreading activation changes the landscape of retrievable memories, retrieval cues systematically restrict the set of potentially retrievable memories, thus reducing competition. This difference in computation is mirrored, to an extent, in the different role played by their putative biological substrates. For example, while the pathways that connect the neocortex to the hippocampus facilitate the retrieval of contextually relevant memories (as outlined in the “Spreading Activation and Cortical Projections to the Hippocampus” section), the VLPFC seems to be involved in reducing the competition between possible retrieved options (Badre & Wagner, 2007; Thompson-Schill et al., 1997). This hypothetical function of the VLPFC as a mechanism to restrict the possible memories competing for retrieval is also consistent with the role of VLPFC in confabulation (Johnson & Raye, 1998). Confabulating patients often make up stories. This could be explained by the lack of retrieval cues (which would restrict the possible set to relevant personal information) combined with the presence of contextual activation. One prediction of this hypothesis is that false memories and confabulations should be induced by any damage that selectively impairs the use of features as retrieval cues. This seems to be the case. For example, while lesions to the ventral prefrontal cortex do induce confabulations, so do lesions along the pathways that connect this region to the hippocampus (including thalamic regions). Another mechanism that can make retrieval cues ineffective is the loss of the specific features used as cues across memories. For example, a retrieval cue of the form (Color: Yellow) would have no effect if there are no memories containing the feature “Color” or its specific value “Yellow”. Indeed, false memories and confabulations increase in individuals with significant temporal lobe and hippocampal neurodegeneration, which would be modeled as a reduction in the number of memories and in the richness of features encoded in each memory (this is consistent, for example, with how Rogers and colleagues model semantic dementia: Rogers et al., 2004).

As noted in the “Retrieval Control, Similarity, and Partial Matching” section, the degree to which features constraints are respected during the retrieval process in ACT-R is modulated by partial matching (Eq. (7)). If the VLPFC control of retrieval occurs through its connection to the hippocampus, then partial matching can be understood as arising from the noisy, distributed activity of these projections (see Fig. 5), allowing for a greater realism in the retrieval process.

Partial matching adds a continuous (dis)similarity dimension to memories, which would otherwise be discrete and separate entities. This allows for semantic memories to be placed within a continuous representational space, as it is, indeed, the case of semantic representations in the human brain (Huth et al., 2012). Most importantly, it allows for memories and their features to be confused and, therefore, to cause retrieval errors even when the retrieval cues would identify a single memory (see Fig. 5). This type of content-based mistakes is an interesting property of autoassociative networks and content-addressable memories Hopfield (1982) that would be otherwise missing in symbolic systems.

## Extensions to ACT-R and Their Relationship to the Neurobiology of Memory

In this section, we will review a number of common extensions to ACT-R and their relationship to the neurobiological mappings discussed above and illustrated in Fig. 5.

### Episodic and Semantic Memory

As noted above, human declarative memory is typically divided into episodic and semantic subsystems (Squire, 2004), but such a distinction is not present in ACT-R. ACT-R’s declarative memory is generally explained as a semantic memory system, although the architecture is agnostic as to whether the information contained in a chunk’s slots is semantic or episodic.

Episodic memory is a person’s spatio-temporal awareness of the events of their own history. As such, it stands to reason that episodic memory utilizes similar neural mechanisms to spatial memory. Ekstrom and Bookheimer (2007) demonstrated that the hippocampus and parahippocampal cortex are preferentially recruited for temporal and spatial-associative retrievals; both key components of episodic memory. Furthermore, Cognitive Map Theory (Burgess et al., 2002) argues for a broader function of the hippocampus including lateralization with the left hippocampus encoding narrative-like linguistic spatial representations and the right hippocampus storing spatial relationships. This information is integrated with temporal information from the frontal lobes to create an analogous “time-stamp” to certain episodic information, which provides the basis for a contextual spatio-temporal episodic memory system.

Functionally, episodic memory provides essential contextual information to prime memory retrieval above and beyond semantic memory alone. For instance, when the episodic context changes between encoding and retrieval, subsequent recall is relatively reduced compared to when retrievals occur in a similar context (Godden & Baddeley, 1975). This episodic context can be both internal (e.g., physiological or mood related: Eich et al., 1994) or external (e.g., environmental: Smith & Vela, 2001). Similar context-dependence has been found for language-dependence (Marian & Neisser, 2000) and motivation-dependence (Delgado et al., 2004) as well.

Some of the present authors’ prior research has shown that without a computational implementation of episodic memory in ACT-R, it was challenging for a model to recall sequences of semantic information in a plausible manner (Thomson et al., 2014). By introducing a richer temporal context from buffer decay, the authors have been able to unify models of free and serial recall (Thomson et al., 2015) as well explain the role of interference in memory effects in memory consolidation (Thomson et al., 2017). Other examples integrating temporal contextual information includes a model of how batters predict baseball pitch speed (Lebiere et al., 2003), and a model of sequence learning (Lebiere & Wallach, 2000).

### Recollection and Familiarity

The ACT-R theory does not distinguish between *familiarity* and *recollection* in making memory judgements; these alternative routes are, instead, incorporated in multiple alternative models (Diana et al., 2007). Extensive imaging evidence suggests that these two alternative mechanisms rely on different neural circuits, with recollection relying on the hippocampus and prefrontal cortex and familiarity relying on regions surrounding the hippocampus (Yonelinas, 2002). Based on these findings, it seems that the neurobiological interpretation presented herein and summarized in Fig. 5 strictly reflects the process of recollection, and does not reflect the memory effects of familiarity. An extension of the ACT-R theory (the Source of Activation Confusion model, proposed by Schunn et al., 1997) allows for familiarity judgment based on the internal perception of the activation of chunks competing for retrieval. In this case, familiarity judgments can be made before or even in absence of successful retrievals, based solely on the activation of available responses.

### Memory and Emotion

Although central to the memory encoding and retrieval system, the hippocampus is also part of a network of anatomical regions collectively known as the limbic system (Rajmohan & Mohandas, 2007), many of which have a prominent role in processing rewards, punishment, and emotion. Among those, the amygdala, a small nucleus located in front of the hippocampus, is perhaps the most prominent. It plays a fundamental role in processing fear and stress (LeDoux, 1998) and, like the hippocampus, it receives widespread cortical projections. The amygdala projects directly to the hippocampus, but does not receive projections from it. Recordings of spontaneous activity at rest suggest that the amygdala is strongly correlated with a number of other regions (including the anterior cingulate cortex) collectively known as the salience network and believed to be responsible for detecting behaviorally important stimuli.

Critically, the amygdala plays an oversized role in modulating memory consolidation in the hippocampus, so that events that trigger amygdala activation are remembered better (McGaugh, 2002; Phelps, 2004). In behavioral experiments in humans, memory for emotional stimuli is typically better than for neutral ones, and their superiority is correlated to the degree to which emotional stimuli activate the amygdala (Dolcos et al., 2004).

The ACT-R theory of memory does not include specific mechanisms to capture these effects. However, several proposals have been made in this direction. Juvina et al. (2018) have proposed an emotion module in which several dimensions of emotion (such as valence and arousal) are added as separate terms to the ACT R equations. These emotional values are learned over time using a reinforcement learning-like rule, in which emotional values associated with a memory are used as predictions and, if the emotional value associated with the re-encoding of the same memory is different, the difference is used to correct the previous estimates.

Smith et al. (2021) have proposed a similar but simpler mechanism. In Smith’s characterization, all dimensions of an emotion are reduced to a single value, the memory’s emotional intensity *I*(*m*), which is similarly modeled as an additive term to the activation equation. Smith et al. (2021) justify this additive term on the basis of the same Bayesian analysis that had originally inspired ACT-R (Anderson, 1990) and is captured in Eq. (1). Specifically, they argued that, from an adaptive decision-making perspective, the baseline probability of retrieving a memory should be the product of its prior probability history and its perceived value, so that the base-level activation of a memory becomes analogous to its overall utility. The additive term, then, comes from the memory activation as an expression of the logarithm of the product of these two terms.

## Discussion

In this paper, we have provided an overview of how the different subcomponents and processes of the ACT-R theory of memory correspond to the known neuroanatomy of memory circuits. This mapping is based on the experience of the authors on various facets of memory. It had never been attempted before to this level of detail.

### Limitations

We recognize that there is a limit to the consistency of the proposed neurobiological mapping. Although ACT-R has incorporated neuroscientific findings in the past two decades of development, both its origins and a large part of its early developments were independent of it. Thus, a number of limitations must be acknowledged.

The first is the semantic network structure of the chunk representations. At the core, this interconnected representation is made possible by the existence of pointer-like structures within a memory, which makes it possible for a memory to directly refer to another one. Nonetheless, this recursive pointing system allows the creation of potentially infinite nested memories. Although some prominent neural architectures (Eliasmith et al., 2012; Rougier et al., 2005) have proposed possible mechanisms by which these symbolic references could be achieved, there is currently no known brain mechanism that allows for their existence.

Another limitation comes from the nature of cortical buffers. In contrast to the widely held assumption that memories are massively distributed representations, in ACT-R, this is not a necessary consequence of retrieval: upon retrieval, a memory is placed in a dedicated, localized buffer where its presence might not affect the contents of any other cortical region. A related issue is that, while memories are believed to be distributed, a single memory in ACT-R can possibly be loaded into a single buffer, therefore assuming a local, cortical representation.

A related limitation is that, in the larger ACT-R architecture, both the delivery of retrieval constraints and the re-instantiation of memory features in the original cortical areas (the “Ghost of brain states past”; Danker et al., 2008) must be mediated by procedural knowledge, which is identified with the basal ganglia (Anderson et al., 2008). The constraint that all forms of voluntary retrieval control and memory re-instantiation must be mediated by the basal ganglia is perhaps one of ACT-R’s most radical departures from the known neurobiology of memory. One possible way to reconcile the two is to relax the bidirectional identification of the architecture’s procedural knowledge with the basal ganglia. In fact, at least one previous study has argued, based on the ACT-R modeling of a neurostimulation experiment, that procedural knowledge incorporates the functional properties of certain cortico-cortical pathways (Rice & Stocco, 2019).

### Predictions

These limitations notwithstanding, both the framework outlined here and the ACT-R theory are specific enough to allow for a number of hitherto untested predictions. In fact, it is somewhat surprising that these predictions have not been tested before.

The first prediction concerns the nature of chunk representation. As noted above, chunks can be thought of as vector representations. If so, chunks that share similar values should have more similar representations than chunks that share different values. That means, it should be possible for a researcher to use multi-voxel pattern analysis (MVPA; Haxby et al., 2001; Mitchell et al., 2008) to decode specific representations from the dedicated regions that are associated with ACT-R buffers (Fig. 4). An MVPA classifier could be trained on a subset of representations, and its performance should degrade in proportion to the predicted number of slots that are shared between the training and the testing items. Alternatively, representational similarity analysis (RSA; Kriegeskorte et al., 2008) could be used to compare the representation between that region’s voxels in two conditions predicted to be associated with different chunks: the degree of similarity between the two representations should vary as a function of different features. Finally, RSA between two regions could be used to provide a ground truth for ACT-R parameters that explicitly describe the similarity metrics between chunks, such as the mismatch penalty *MP* or the dissimilarity metric δ in Eq. (7). Two specific buffers seem particularly well qualified for this experiment: one is the “retrieval” buffer and the other one is the “imaginal” problem-state buffer (Borst et al., 2010).

As Fig. 5 shows, some of the computations of the ACT-R theory are tentatively mapped onto the pathways that connect cortical regions to other cortical and subcortical areas. This allows for testing potential *causal* interventions. For example, applying Transcranial Magnetic Stimulation (TMS) of cortical regions should have different effects during retrieval of information. Specifically, TMS of the parietal regions should disrupt spreading activation, making the retrieval of contextually-relevant information harder. TMS of the lateral prefrontal region (the retrieval buffer) should systematically affect the constraints of the retrieval. To the best of our knowledge, only one study so far (Rice & Stocco, 2019) has ever used TMS to investigate the nature of memory representations in ACT-R and, although that study focused on *procedural* rather than declarative knowledge, its encouraging results show that the approach is viable.

The role of cortical and subcortical connectivity may also be examined using functional connectivity of neuroimaging data. In this type of analysis, correlations between the time series of the BOLD signals in two different regions are taken as a measure of the degree of information that is being exchanged between the two. In ACT-R terms, the degree of correlation could be related to either the amount of spreading activation or, in the specific case of the lateral prefrontal cortex, the degree of influence affected by the retrieval specification and constraints or the mismatch penalty *MP* in Eq. (7).

Finally, a tantalizing possibility is that stable individual differences in behavior (that is, psychometric *traits*) can be related to individual differences in parameters, as first proposed by Gobet and Ritter (2000). Evidence in this sense comes from a few studies. For example, Daily et al. (2001) found that individual differences in the *W* parameter of Eq. (6) were related to individual differences in working memory capacity. Recently, Sense et al. (2016) and Hake et al. (2023) found that individual differences in the α parameter of Eq. (5) are stable across sessions and materials and are associated with performance in neuropsychological tests of long-term memory function. It is possible, therefore, that individual differences in the parameters of ACT-R’s memory equations are related to measurable differences in the function of these circuits. For example, individual differences in the *W* parameter should be related to individual differences in the connectivity between parietal cortices and the hippocampus. Connectivity could be measured directly using water diffusion tractography techniques or, as it is more common, using resting state functional connectivity, i.e., the degree of correlation between the spontaneous activity of two regions at rest.

### Concluding Remarks

It is our hope that this mapping would provide a useful bridge between two communities: cognitive neuroscientists studying memory and cognitive and computational scientists using and developing cognitive models. Because it provides a mapping between concepts used within these communities, we think of this paper as a dictionary that can be used in two ways. First, it provides a tool for neuroscientists to understand how relevant processes can be formalized and approximated in an existing and popular computational framework. Second, it provides a way to interpret computational mechanisms and parameters in a biological way. This latter use could be helpful in guiding model development, deciding, for instance, whether a particular extension of the theory is biologically plausible or warranted, or how changes in parameter values could be interpreted.

We should stress that this proposed mapping is speculative and largely driven by the knowledge and research experience of the authors; as we have acknowledged above, the mapping is certainly limited and approximate in many aspects. Nonetheless, the suggested biological interpretation of ACT-R’s memory framework is sufficiently precise that a number of experimental predictions can be derived, and further experimental work could either confirm or refine the proposed mappings and biological substrates. In fact, the opportunity to provide a roadmap for such future experimental work constitutes a third, and notable, goal of this paper.

## Footnotes

1 Note that a feature’s name is only a notational convention, and has no implication for how memories are implemented in the brain.

2 Note that this term does not capture, however, the more “active” forms forgetting due control processes, such as motivated forgetting (Anderson et al., 2008) or retrieval-induced forgetting (Anderson & Bjork, 1994).

## Declarations

### Disclaimer

The views and conclusions contained in this document are those of the authors and should not be interpreted as representing the official policies, either expressed or implied, of the U.S. Government.
